# Unethical Organization Behavior: Antecedents and Consequences in the Tourism Industry

**DOI:** 10.3390/ijerph19094972

**Published:** 2022-04-20

**Authors:** Ibrahim A. Elshaer, Alaa M. S. Azazz, Samar K. Saad

**Affiliations:** 1 Department of Management, College of Business Administration, King Faisal University, Al-Ahsaa 380, Saudi Arabia; 2 Hotel Studies Department, Faculty of Tourism and Hotels, Suez Canal University, Ismailia 41522, Egypt; 3 Department of Tourism and Hospitality, Arts College, King Faisal University, Al-Ahsaa 380, Saudi Arabia; 4 Tourism Studies Department, Faculty of Tourism and Hotels, Suez Canal University, Ismailia 41522, Egypt; samar_kamel@tourism.suez.edu.eg

**Keywords:** unethical organization behavior, work intensification, job insecurity, unethical company-profit climate, feeling of guilt, emotional exhaustion, customer-oriented citizenship behavior

## Abstract

The entire tourism and hospitality industry has witnessed a considerable increase in the number of ethical difficulties that occur in the workplace. It has been discovered that unethical organizational behavior (UOB) is the most significant category in tourists’ unpleasant experiences, driving them to switch and spread unfavorable word-of-mouth information. This study aims to explore the effects of three contextual factors on UOB (i.e., work intensification, job insecurity, and an unethical company-profit climate) and to investigate its possible employee-related consequences, including the feeling of guilt, emotional exhaustion, and customer-oriented citizenship behavior. A total of 970 employees working in hotels (5-star and 4-star) and travel agencies (Category A) participated, and the obtained data were analyzed by structural equation modeling. The results asserted that work intensification, job insecurity, and an unethical company-profit climate stimulate unethical organizational behavior, and unethical organizational behavior leads to feelings of guilt, emotional exhaustion, and customer-oriented citizenship behavior. Significant insights into theoretical and practical implications were further discussed.

## 1. Introduction

The significant rise of ethical challenges in the workplace has been evident to the entire tourism and hospitality industry. According to the Report to the Nations, a global study on occupational fraud and abuse published in 2020 [1], the total cost that foodservice and hospitality organizations lose because of unethical behaviors sums up to approximately 5% to 6% of annual revenues. Unethical business practices have been also found to represent the most important category in tourists’ negative experiences, causing switching and negative word-of-mouth [2]. Although most tourism and hospitality organizations have set up standard operation procedures, such procedures are not followed in reality [3]. Law and professional code do not seem to guarantee the ethical choice of employees.

The organizational context probably plays a fundamental role in distorting ethical decision making leading to unethical behavior; however, this issue has drawn little attention from hospitality researchers [4]. Most research that investigated the ethics within the hospitality workplace context has focused on ethical perceptions and values [3,5], social [6] and environmental responsibility of organizations [7], and ethical incident classification [8]. Relatively few studies have discussed the predictors of unethical organizational behavior (UOB) in the tourism and hospitality workplace context [9,10]. This is surprising because it is generally suggested that UOB should result in severe outcomes, such as the overall decline of organizational performance due to customer turnover [11], and adverse attitudes/behaviors of employees [12]. To address this research gap and based on the conservation of resources theory and the appraisal theory of emotions, this study provides answers to uncovered research questions to identify not only the possible antecedents but also the consequences of UOB in the tourism and hospitality workplace context.

This study is relevant for many reasons. Especially after the COVID-19 pandemic, the tourism and hospitality industry is facing significant challenges. Due to the severe decline in demand and revenues, most companies had to make structural and operational changes (e.g., downsizing and work overload on the remaining employees) [13]. In this context, employees face high levels of stress due to job insecurity, overwork, and the need to keep their organization alive during the pandemic. Workplace stressors may cause employees to engage in UOB to achieve job goals. Identifying such contextual stressors is critical for both researchers and practitioners [4]. Accordingly, this study aims to explore the effect of three contextual factors on UOB (i.e., work intensification, job insecurity, and unethical company-profit climate) and to investigate its possible employee-related consequences, including the feeling of guilt, emotional exhaustion, and customer-oriented citizenship behavior. The study structure started with a theoretical background to build the research framework and justify the research hypotheses, followed by the research methodology in which the study measures were introduced, and the sampling and data analysis techniques were explained. The study results were then discussed, and theoretical and practical implications were elaborated. Finally the study ended with limitations and further research opportunities and the conclusion section.

## 2. Theoretical Background and Hypotheses Development

Scholars have largely studied UOB as a purely self-serving activity that aims to satisfy an individual’s selfish interests and results mainly in terms of personal gain [14]. Umphress, Bingham, and Mitchell [15] advanced that perception and suggested that UOB may extend beyond the self-focused views and include employees’ actions that aim to benefit their organization or group (e.g., harming rivals or withholding information to boost the organization’s status in the market). Individuals may participate in such actions that contravene social values, laws, or proper conduct because they believe that this would support their organization and benefit them accordingly [16].

Academics have conceptualized UOB from two sides. First, it is unethical because it violates social norms or standards of ethical behavior [17]. Therefore, work-related actions that involve mistakes or unconscious negligence are not considered UOB. Second, it is pro-organizational because such behavior is not instructed by leaders or supervisors nor included in formal job description; however, it is voluntarily conducted to satisfy the interests of the organization or its members [15].

The organizational environment may provide a stimulus or a deterrent for UOB. From the perspective of UOB itself, employees act unethically if they feel the need to do that or it makes sense that it is appropriate to behave that way. This means that their workplace environment and its features can trigger their UOB. The results suggest that individuals who suffer from stress and strain due to workplace pressure or threats may practice UOB as a way to retain their job stability. Workplace pressure, such as the depletion of valuable resources, makes employees work under adverse conditions and accordingly experience stress. According to the conservation of resources theory [17], when people face circumstances that result in fear of losing valuable resources, they experience stress that pushes them to protect such resources. They reobtain the balance by defending, conserving, and acquiring recovery resources. Therefore, the study results suggest that threats of losing valuable resources such as time and energy due to work intensification, total job or job features, and the company’s financial resources may motivate employees to act unethically to protect their assets.

On the other hand, people strive to maintain, boost, and protect their self-esteem [18]. Acting immorally and harming others (e.g., customers or competitors) can distort the beliefs that employees think about themselves, cause drops in self-esteem, and activate self-relevant emotions. According to Teng et al. [12], these emotions can be positive if individuals translate their UOB as beneficial for the organization or be negative if individuals see that they violated ethical standards and harmed others. According to the appraisal theory of emotions [19], individuals’ cognition controls the type of emotions they would feel after having a specific attitude or behavior. Individuals appraise their current state positively or negatively according to their cognitive self-representation. Drawing on the appraisal theory of emotions, it can be assumed that employees would experience feelings of guilt and emotional exhaustion as negative emotions after appraising their unethical behavior and engage in practices that may enhance their self-esteem, such as customer-oriented citizenship behaviors.

### 2.1. Job Insecurity as an Antecedent of UOB

Job insecurity, which is defined as employees’ concern about the future continuance of their jobs [20], has been found to have a series of relationships with job preserving-related outcomes [21]. It triggers employees to act in ways that they believe might protect them from losing their job or job feature. Because most organizations associate continuous employment with performance, employees might strive to demonstrate their valuable contribution to the organization. They devote extra effort towards behaviors that will be noticed and valued, specifically when they face exclusion risk. Such effort may be in the form of UOB [16].

**Figure 1:**
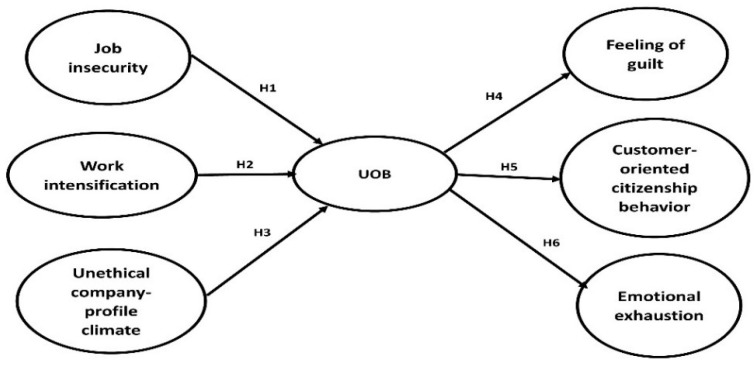
Research framework.

Although there is evidence that employees may engage in UOB to keep their job or job features stable [22,23], the outcome is not for the sake of the organization. Past research was conducted in work contexts where the source of job insecurity was the organization itself. The UOB was mainly performed to benefit self-interest rather than others (e.g., hiding information from coworkers), probably causing high costs to organizations. Employee responses to job insecurity that are attributed to factors beyond the organization’s control are still to be explored. In this research, the current study takes advantage of the COVID-19 adversity and examine whether the feeling of external threat would lead employees to benefit their organization and help it to survive even by ignoring ethical issues. Therefore, as shown in Figure 1, the below hypothesize can be proposed:

**Hypothesis** **1** **(H1).** *Job insecurity is associated with UOB*.

### 2.2. Work Intensification as an Antecedent of UOB

Most managers put more pressure on their employees to work faster and under tighter deadlines, multitask, and reduce their idle time to gain a competitive advantage or to cope with changes in work situations [24]. Such work intensification represents a typical challenge stressor and affects work outcomes, for example, thriving at work [25], turnover intention, and burnout [26]. Recent organizational and technological changes associated with the COVID-19 pandemic have significantly contributed to the pressure of work intensification [26]. Downsizing—which leads to restructuring organizations and accelerating workloads on remaining employees—is a significant driver of work intensification. Employees need to work long or unsocial hours to cover the growing number of tasks that their laid-off fellows used to perform [13].

Work intensification has been associated with intended low quality of service performance (an example of UOB) in the hospitality industry [27,28]. For example, room attendants have been found to perform a fast-paced room cleaning, which was associated in some cases with low quality, especially when they are paid on a piece-rate (the number of rooms cleaned) [28]. This is reasonable, as maintaining quality service often involves exerting extra effort, spending more time, and/or using excessive organizational resources. Therefore, employees who experience work intensification may perform UOB to make a balance between their organizational requirement and their own requirement, wishing that doing so would increase the chances of gaining appropriate wages and acceptance in their organization. Therefore, the following hypothesize can be suggested:

**Hypothesis** **2** **(H2).** *Work intensification is associated with UOB*.

### 2.3. Unethical Company-Profit Climate as an Antecedent of UOB

The ethical organizational climate is defined as the shared norms in an organization regarding the correct behaviors and how ethical issues should be practiced within the organization [29]. The ethical climate can be perceived in organizations within three criteria as classified by Victor and Cullen [29]: individual, local, and cosmopolitan level. Hospitality empirical research has suggested that the local level, which focuses on company profit, team interest, and company rules and procedures, has the most influence on the ethical perception when making decisions [8,30].

In this study, we focus on the correlation between the unethical company-profit climate and UOB in organizations. The unethical company-profit climate is perceived when individuals’ decisions represent the organization’s best interests regardless of the consequences [14]. Hospitality research has demonstrated many examples of that context. For example, [7] found that restaurant managers avoid investing in environmentally-friendly practices despite being informed and concerned about the environmental issues due to financial and operating concerns [7]. In the same vein, [6] reported that tourism marketers of larger organizations tend to less consider the social responsibility of their organizations. The authors in [3] also stated that although hoteliers clearly recognize ethical issues in the workplace, they are more likely to participate in unethical behavior to increase income, such as not stopping drunk customers from ordering alcohol. Therefore, the below hypothesize is introduced:

**Hypothesis** **3** **(H3).** *An unethical company-profit climate is associated with UOB*.

### 2.4. Feelings of Guilt as an Outcome of UOB

Work behaviors that reflect individuals’ inability to meet their goals/expectations or personal/organizational standards probably activate a self-evaluative process that consists of re-thinking the behavior, the conflict resulting from it, or the unethical decision one has made [31]. Signals that individuals are acting immorally (e.g., lying or cheating) may result in deep self-conscious feelings such as self-blame of the inability to fulfill one’s ethical standards [32] and guilt [33]. Participating in UOB can make individuals perceive that their behavior that violated moral standards has distorted their self-image. For example, Goh and Jie [5] found that generation Z hotel workers feel guilty about their food wastage behaviors. They believe that this act is unethical because it is bad for the environment and a waste of money. However, they are inclined to do it to comply with the management policy that targets customer satisfaction through fresher produce. Similarly, Tang et al. [12] found that customer services agents often feel guilty after engaging in UOB with customers (e.g., misrepresenting the truth to make their organization looks good). Therefore, the following be hypothesized:

**Hypothesis** **4** **(H4).** *UOB is associated with feelings of guilt*.

### 2.5. Customer-Oriented Citizenship Behavior as an Outcome of UOB

Customer-oriented citizenship behavior refers to the discretionary behaviors that service employees can do toward customers and go beyond their formal role [34,35]. These behaviors can include providing customers with extra service quality and helping them after hours or off duty. This study suggests that employees who practiced UOB may tend to compensate the affected customers by providing them with additional help or service than what is usually formally provided. Previous research has suggested a correlation between citizenship behavior and some other constructs that are close to UOB. For example, [36] found that when individuals are made aware of their high level of counterproductive behavior towards their organization and informed that their behavior is not appropriate, they do compensatory behaviors such as organizational citizenship behaviors. Similarly, [8] suggest that service employees may feel guilty for behaving unethically with the customers (e.g., cheating or hiding knowledge), therefore engage in a self-enhancing behavior or self-compensatory behavior. Therefore, the below hypothesize is suggested:

**Hypothesis** **5** **(H5).** *UOB is associated with customer-oriented citizenship behavior*.

### 2.6. Emotional Exhaustion as an Outcome of UOB

Emotional exhaustion is the sense of being overextended and drained by one’s job [37]. Individuals who are emotionally exhausted usually feel physical fatigue and lack energy. This study suggests that individuals who practice UOB may experience feelings of emotional exhaustion. The practice of UOB can either be occasional or more constant; however, even brief engagement to UOB results in a stressful experience [32]. People usually strive to perceive themselves and convince others that they are worthy and respectful. Therefore, practicing unethical behavior threatens their self-esteem, as they attribute their unethical behavior to their lack of moral strength. This causes them to feel salient stress. However, according to Crocker and Park [38], individuals can overcome such threats and stress by finding ways to maintain balance. For example, employees may try to enhance their self-representation by appraising themselves as good workers who benefit their organization rather than bad ones who cheat customers [12]. However, the uncontrollability of the situations following UOB and the constant pressure to continue practicing such behavior probably creates feelings of emotional exhaustion. Therefore, the following hypothesize is proposed:

**Hypothesis** **6** **(H6).** *UOB is associated with emotional exhaustion*.

## 3. Research Methods

### 3.1. Measures

All factors in this study were obtained from previously published scales as a result of extensive literature reviews. Umphress et al.’s [15] 7-item multi-dimensional scale for unethical organization behavior was employed. An example item is, “If it would help my organization, I would misrepresent the truth to make my organization look good.” Job insecurity was measured by 6 items derived from [39]. An example item is, “I am afraid I may lose my job shortly.” Employees responded to a three-item scale measuring feeling of guilt established by [40] and used by [41] to express their level of guilt. Participants expressed their agreement with adjectives describing their feelings during job contacts with consumers. “Guilty” is an example. Similarly, the intensification of job demands scale (5 items) developed by [24] was employed to measure work intensification. Additionally, the study utilized the Victor and Cullen [29] and Cullen et al. [42] ethical climate scale, which includes three items that show egoistic self-interest behavior as unethical company climate behavior. Employees reported their level of guilt with the 3-item scale developed by [40], and used by [41]. Participants reported their agreement with adjectives about how they felt in interactions with customers at work.” Employees completed a 5-item measure designed by Bettencourt and Brown [34] to assess their customer-oriented citizenship behavior (OCBC). An example variable is, “Today, I helped customers with problems beyond what is expected or required.” Finally, emotional exhaustion was measured by six variables developed by Karatepe and Uludag [43], based on Maslach and Jackson’s [44] study, and implemented on hotel employees located in Northern Cyprus. Respondents were asked to rate their level of agreement with each statement on a five-point Likert scale (1 indicating strong disagreement, and 5 indicating strong agreement).

### 3.2. Data Collection and Sampling Characteristics

To collect data, a self-administered survey was developed. The data for this paper were obtained randomly from employees at five- and four-star hotels, as well as travel agents classified as category A. Twenty-five student enumerators from public colleges in Egypt were trained to collect data from the targeted employees in greater Cairo (the country’s largest metropolitan region). This methodology was chosen to circumvent the frequently poor response rate associated with traditional postal and/or online data collection methods [45,46] and to circumvent respondents’ unwillingness to answer the survey [47]. During the data gathering procedure, enumerators took healthy steps to protect themselves and others from infection amid the COVID-19 pandemic.

Prior to participating in the study, respondents signed a consent form. Enumerators were taught to read the questionnaire in plain language and record respondents’ responses. A total of 1000 responses was collected, and 30 questionnaires were discarded due to insufficient responses, leaving 970 acceptable surveys for analysis. Data were collected between May and June of 2021. The current study sample size was 970, and it met Nunnally’s [48] suggestions for SEM testing of ten cases per item. The study scale had 35 items; hence, the current sample size (970) exceeded the recommended 350 responses (10 × 35). The sample size of 970 was satisfactory as recommended by Boomsma’s [49] based on the ratio of variables (*p*) to dimensions (k), which is 5.93 (35 observed indicators/six latent constructs), requiring 200 responses as a minimum number of sample size. Furthermore, 970 responses fulfilled the criteria suggested by Hair et al. [50] for 100 to 150 minimum sample size to achieve a satisfactory result. Finally, while Krejcie and Morgan [51] suggested a minimum sample size of 384 responses if the total population exceeded 1,000,000, the current study obtained a sample size of 970, exceeding the recommendations. 

The mean differential scores for early and late responses were analyzed using the independent sample *t*-test method. There were no statistically significant differences between early and late responses (*p* > 0.05), indicating that non-response bias was not an issue in this study [52].

As depicted in Table 1, 495 (51%) of the 970 responders were hotel employees, whereas 475 (49%) were travel agent employees. The majority (60%) were male and married (73%). Over half (59%) of respondents were between the ages of 30 and 45. Around 60% were graduates of a university. In terms of tenure, 281 respondents (about 29%) had worked for their organization for less than five years, while 505 respondents (52%) had worked for their organization between six and fifteen years. The mean scores (M) ranged between 3.71 and 4.15, while the standard deviation (S.D.) values ranged between 0.726 and 1.077, indicating that the data were more dispersed and less marked around the mean [53]. The values of skewness and kurtosis (data distribution) were not greater than −2 or +2, indicating that the data had a normal distribution, as shown in Table 2 [54].

### 3.3. Data Analysis

SPSS vs25 was used to determine the respondents’ descriptive characteristics, conduct the independent sample *t*-test, and assess the study dimensions’ reliability using Cronbach’s alpha scores. Due to the complexity of the suggested model, the current study explored its structural properties using confirmatory factor analysis (CFA) and structural equation modeling (SEM) with AMOS vs20.

## 4. Results

### 4.1. Measurement Model

**Figure 2:**
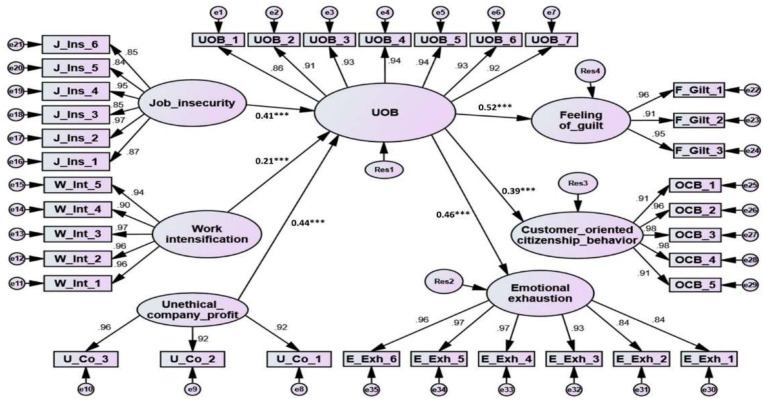
Structural and measurement model. ***: significant level less than 0.001.

To assess the validity and reliability of the study measurement model, first order CFA with Amos vs20 was used. As illustrated in Table 2 and Figure 2, the model exhibited a high degree of data fit: χ^2^ (539, N = 970) = 1763.069, *p* < 0.001, normed χ^2^ = 3.271, root mean square error of approximation (RMSEA) = 0.031, standardized root mean squared residual (SRMR) = 0.029, comparative fit index (CFI) = 0.954 (Table 2).

All Cronbach’s alphas and CR scores were above the recommended 0.80 level [55], which indicated high internal consistency and reliability. All standardized loadings, as shown in Table 2, ranged from 0.840 to 0.978, which exceeded the acceptable value of 0.7, with t-values above 33.990 [56] (Table 2). This showed a significant positive relationship between the study dimensions. Hence, convergent validity was guaranteed. The Average Variance Extracted (AVE) values (as shown in Table 2) for all utilized factors exceeded the suggested 0.50 threshold [55], further confirming and supporting convergent validity.

Two methods were used to assess discriminant validity. To begin, the AVE square root of each dimension should be greater than the values of the common correlations between the dimensions in row and column [55]. Second, Hair et al. [50] proposed that for discriminant validity, the AVE should exceed the dimension’s maximum shared value (MSV). As seen in Table 2 and Table 3, the AVE scores exceeded the MSV, and all items were more heavily loaded on their factor than on any other, indicating item-level discriminant validity. In other words, the results established the measurement model’s reliability and validity.

### 4.2. The Structural Model

To examine the causal relationships between research variables, structural equation modeling (SEM) and maximum likelihood estimates were used. SEM is an appropriate data analysis approach because it enables the concurrent and inclusive study of proposed relationships [55]. In general, the structural model’s fit indexes χ^2^ (554, N = 970) = 1865.872, *p* < 0.001, normed χ^2^ = 3.368, RMSEA = 0.038, SRMR = 0.047, CFI = 0.929, TLI = 0.931, NFI = 0.942, PCFI = 0.736 and PNFI = 0.731) show a satisfactory model fit (see Table 4).

### 4.3. Testing Research Hypothesis

Table 4 and Figure 2 display the interrelationships between the research variables. The results suggest that job insecurity had a positive and significant relation with UOB (β = 0.41, t-value = 12.187, *p* < 0.001), and accordingly, hypotheses H1 was supported. Similarly, work intensification was found to have a positive and significant impact on UOB (β = 0.21, t-value = 7.226, *p* < 0.001), which supports hypotheses H2. The Amos results gave signals that unethical company profit climate positively and significantly impacted UOB (β = 0.44, t-value = 14.142, *p* < 0.001); accordingly, hypotheses H3 was supported. Furthermore, UOB was found to have a positive significant impact on feelings of guilt (β 0.52, t-value = 17.00, *p* < 0.001), customer-oriented citizenship behavior (β 0.39, t-value = 10.204, *p* < 0.001), and emotional exhaustion (β 0.46, t-value = 14.448, *p* < 0.001); hypothesis H4, H5, and H6 were therefore accepted.

## 5. Discussion and Implications

This study, which investigated the antecedents and consequences of UOB in a tourism and hospitality workplace context, has significant findings. Overall, drawing on the conservation of resources theory and the appraisal theory of emotions, the results supported the model and suggested that UOB is linked to workplace scarcity of resources and generates employees’ subsequent emotional reactions. Threats of reduction in workplace resources such as time, energy, the loss of job/job features, and lack of financial resources increase employees’ risk-taking and trigger them to engage in UOB. Their unethical actions are guided by beliefs that engaging in UOB would promote organizational functioning and profits and enable them to regain such resources back. Acting unethically, in turn, causes a drop in employees’ self-esteem, which causes a rise of negative emotions (i.e., guilt and emotional exhaustion) and prompts employees to engage in positive behaviors (i.e., customer-focused citizenship behavior) to wash away their feeling of shame.

On the other hand, even though the negative outcomes of UOB have been seen to be obvious, this belief is relatively shallow. According to [12], UOB is followed by not only negative employees’ emotions and behaviors but also positive ones that still need more research. The lack of empirical research and, accordingly, the lack of detailed indications eliminate organizations’ abilities to appropriately manage UOB and its consequences and generate desired results. Against this backdrop and taking advantage of the work stressors generated by the COVID-19 pandemic, this study gives evidence that three contextual factors (i.e., work intensification, job insecurity, and unethical company-profit climate) have a direct positive influence on UOB, and in return UOB has three possible employee-related consequences, including feelings of guilt, emotional exhaustion, and customer-oriented citizenship behavior. The study has several theoretical and practical implication as follows.

### 5.1. Theoretical Implications

These results have valuable theoretical implications. First, observed support was found for a UOB model in a tourism and hotel workplace context. While the hospitality literature has made recent efforts to investigate perceptions of UOB incidents [5,6] and possible strategies to attenuate it [9,10], this research extends our understanding by developing and testing a parsimonious yet robust model of the antecedents and consequences of UOB. The study showed the roles work intensification, job insecurity, and an unethical company-profit climate play in influencing employees’ engagement in UOB and the effects these behaviors have on employees’ self-esteem.

More importantly, most prior research on UOB was built upon social influences theories such as the theory of social identity, theory of social learning, and social cognitive theory (see the systematic review on UOB research by Mishra et al. [55]). Such studies discuss how employees’ behavior is influenced by good and bad workplace exemplars (e.g., leadership and corrupted colleagues). Yet, it seems that researchers have overlooked a critical component of the UOB phenomenon: the interrelation between organizational contextual resources and employees’ needs and consequent behaviors. Departing from the mainstream study approach, the study examined the antecedents of UOB based on the conservation of resources theory. The study provides significant evidence that employees who experience a lack of resources within their workplace are more inclined to participate in UOB to alleviate this state.

Second, the study addressed the lack of research into the organizational context antecedents to UOB in hospitality research [4]. Our results provide evidence that work intensification (work overload and time pressure) increases unethical behavior. This finding raises possible concerns about unintended outcomes of commitment to impractical goals. Work intensification probably enhances the likelihood that employees perform their tasks with disregard of ethical standards.

The results also reveal the significant relationship between feelings of job insecurity—resulting from factors beyond the organization’s control—and UOB. Consistent with past literature [10,22], individuals who experience job insecurity probably feel the need to demonstrate their values to their organization to avoid being laid off or losing important job features. It appears that the pressure of uncertainty and the inability to cope with unexpected future loss trigger the employees to behave unethically for the benefit of the organization and to protect their current jobs.

The study also validates the direct effect of the unethical company-profit climate on UOB. Individuals are more likely to participate in UOB to increase organizational profit when they perceive a widespread acceptance of unethical actions within their organization. In a climate with a scarcity of financial and ethical resources, employees are probably more impulsive, risk-seeking, and focused on maximizing or regaining profit regardless of the social or business norms.

Third, the results reveal strong links between UOB and employees following negative emotions. These findings support Tang et al.’s [12] suggestion that unethical behavior may influence employees’ self-appraisal leading to their feelings of guilt. People are more likely to feel less respect for themselves and shame after engaging in unethical behavior.

The study findings also confirmed that UOB significantly influences employees’ emotional exhaustion. Specifically, the results reveal that employees who take the risk of engaging in UOB and are aware of the harm caused to others due to that behavior are prone to feeling an increased level of emotional exhaustion. Quite similar results are in a study by Meier et al. [32], where UOB was significantly related to stress.

Finally, the findings reveal a strong relationship between UOB and customer-oriented citizenship behavior. This is consistent with findings in [12] that UOB—though being a cause of stress and negative feelings—can produce positive behaviors. It seems that employees engage in customer-oriented citizenship behavior as a way to cope and recover from negative self-appraisal and the following adverse emotions.

### 5.2. Practical Implications

This study has further practical implications for the tourism and hotel industry. First, by investigating the antecedents of UOB, the study provides managers with valuable insights into ways to control and attenuate UOB. For example, managers can reduce workload and train employees to work more efficiently. Managers should also promote a high standard of ethical conduct among employees by emphasizing the consequences of positive or negative behaviors. Regarding job insecurity, managers should seek to make their workplace environment supportive for employees who feel uncertainty due to possible job loss. An environment with transparent communication is very crucial for employees when the organization is considering a downsizing strategy.

Second, the findings suggest that engaging in UOB increases employees’ participation in customer-oriented citizenship behavior. Although engaging in reparative behavior to compensate harmed customers is recommended, UOB is not. Evident from the current results are the negative consequences of UOB on the wellbeing of employees (i.e., feelings of guilt and emotional exhaustion), which according to Grobelna [27] and Kim [31] lead to a series of negative self-conscious emotions, low quality of service performance, and intention to quit. Moreover, past research has asserted the negative outcomes of UOB on the long-term performance of organizations [57]. Therefore, despite the positive consequences of UOB, managers should not accept such unethical behaviors for the benefit of the organization. They should carefully observe the activities of the employees, regularly remind them of the code of ethics, and punish those who participate in such unethical behavior.

## 6. Limitations and Areas for Further Research

This study has several limitations and suggests more research avenues. First, the study investigated three antecedents (work intensification, job insecurity, and unethical company-profit climate) and three consequences (feelings of guilt, emotional exhaustion, customer-oriented citizenship behavior) of UOB; however, other variables can be further explored as consequences of UOB, such as employee performance, company performance, company image and reputation, and job satisfaction, while other factors can be further examined as antecedents of UOB, such as family financial pressure and social disengagement. Second, due to the cross-sectional character of the data, causal correlations between variables cannot be established precisely. Third, while this study focused on preventing CMV in accordance with recommendations of [58], future researchers may employ longitudinal data or a combination of data sources to validate the study’s proposed model. Fourth, the suggested model can be utilized to evaluate these relationships in a variety of contexts (industry or country) by applying a multi-group analysis method [59].

## 7. Conclusions

A significant increase in the number of ethical difficulties that occur in the workplace has been observed across the entire tourism and hospitality industry. Tourists’ unpleasant experiences have been found to be dominated by unethical organizational behavior (UOB), which causes tourists to change destinations and spread negative word-of-mouth information. Organizational context may act as a stimulant or deterrent to UOB. From UOB’s perspective, employees act unethically if they feel compelled to do so or believe that such behavior is appropriate. This means that their work environment and its characteristics may act as a trigger for their UOB. Most research on workplace ethics in hospitality has focused on ethical perceptions and values, organizational social and environmental responsibility, and ethical incident classification. Few studies have examined predictors and consequences of unethical organizational behavior in the tourism and hospitality industries. To fill this research gap, this study examines the antecedents (i.e., work intensification, job insecurity, and an unethical company-profit climate) and consequences (i.e., feelings of guilt, emotional exhaustion, and customer-oriented citizenship behavior) of UOB in the tourism and hospitality workplace. Data were obtained from 970 employees working in four- and five-star hotels and with category A travel agents. The scale’s convergent and discriminant validity was obtained by conducting first order confirmatory factory analysis. The research hypotheses were tested by SEM, and the findings revealed that UOB is associated with a lack of resources at the workplace, which causes employees to experience emotional reactions as a result. Workplace resources such as time, energy, loss of job features, and a lack of financial resources are threatened by reductions in these resources, which increases employees’ risk-taking and prompts them to participate in UOB. Furthermore, employees’ unethical actions are guided by the belief that engaging in UOB will promote organizational functioning and profits, as well as provide them with the opportunity to reclaim those resources. When employees act unethically, their self-esteem suffers, which leads to a rise in negative emotions (such as guilt and emotional exhaustion), which prompts them to engage in positive behaviors (such as customer-focused citizenship behavior) in order to wash away their feelings of shame. Theoretical, this research contributes to our understanding of UOB by developing and validating a simple yet robust model of its antecedents and consequences of UOB in the context of tourism and the hospitality industry. Practically, managers should encourage their employees to maintain a high level of ethical behavior by emphasizing the antecedents and consequences of either positive or negative unethical workplace behaviors.

## Figures and Tables

**Table 1:** Respondents’ characteristics.

		*n* = 970	%
Type of business	Five- and Four-star hotels	130 (495 employees)	51%
	Category A Travel agent	110 (475 employees)	49%
Gender	Male	582	60%
	Female	388	40%
Marital status	Married	708	73%
	Unmarried	262	27%
Age	Less than 30 years	204	21%
	30 to 45 years	572	59%
	45 to 60 years	116	12%
	More than 60 years	78	8%
Education level	Less than high school degree	146	15%
	High school degree	242	25%
	University graduate	582	60%
Years of experience	1 to 5 years	281	29%
	6 years to 10 years	291	30%
	11 years to 15 years	214	22%
	More than 15 years	184	19%

**Table 2:** Results of CFA—*M* and standard deviation.

Factors and Items	Standardized Loading	t-Value	M	S.D.	Skewness	Kurtosis
**UOB (Umphress et al., 2010) (a = 0.971) (CR = 0.977, AVE = 0.858, MSV = 0.281)**						
“If it would help my organization, I would misrepresent the truth to make my organization look good.”	0.863	b	4.10	0.961	−1.544	2.615
“If it would help my organization, I would exaggerate the truth about my company’s products or services to customers and clients.”	0.913	41.845	4.15	0.793	−0.890	0.672
“If it would benefit my organization, I would withhold negative information about my company or its products from customers and clients.”	0.931	43.688	4.10	0.896	−1.274	1.942
“If my organization needed me to, I would give a good recommendation on the behalf of an incompetent employee in the hope that the person will become another organization’s problem instead of my own.”	0.954	46.288	4.12	0.896	−1.340	2.010
“If my organization needed me to, I would withhold issuing a refund to a customer or client accidentally overcharged.”	0.952	46.059	4.11	0.901	−1.347	2.020
“If needed, I would conceal information from the public that could be damaging to my organization.”	0.940	44.699	4.11	0.900	−1.341	2.011
“I would do whatever it takes to help my organization.”	0.927	43.199	4.12	0.886	−1.544	2.615
**Job Insecurity (Hellgren et al., 1999) (a = 0.957) (CR = 0.958, AVE = 0.794, MSV = 0.283)**						
“I am worried that I will have to leave my job before I would like to”.	0.873	b	3.72	0.917	−0.602	0.360
“I worry about being able to keep my job”.	0.972	50.146	3.81	0.874	−0.830	0.647
“I am afraid I may lose my job shortly”.	0.851	36.848	3.90	0.918	−0.698	0.207
“I worry about getting less stimulating work tasks in the future”.	0.949	47.049	3.74	0.889	−0.741	0.469
“I worry about my future wage development”.	0.843	36.168	3.83	0.915	−0.571	0.027
“I feel worried about my career development in the organization”.	0.849	36.723	3.71	0.997	−0.809	0.382
**Work intensification (Kubicek et al., 2015) (a = 0.907) (CR = 0.978, AVE = 0.901, MSV = 0.462)**						
It is increasingly rare to have enough time for work tasks	0.963	b	3.93	1.052	−0.995	0.388
It is increasingly harder to take time for breaks	0.964	77.450	3.89	1.073	−1.014	0.432
The time between the more intense work phases has decreased.	0.968	80.149	3.88	1.082	−0.950	0.210
One has more often to do two or three things at once (such as eating lunch, writing emails, and talking on the phone)	0.904	55.827	3.89	1.073	−1.014	0.432
Ever more work has to be completed by fewer and fewer employees	0.945	68.729	3.87	1.077	−0.949	0.245
**Unethical company-profit climate (Victor and Cullen, 2008; Cullen et al., 2003), (a = 0.951) (CR = 0.953, AVE = 0.870, MSV = 0.335)**						
In this company, people are mostly out for themselves	0.919	b	4.18	0.734	−0.931	1.765
People in this organization are very concerned about what is best for themselves.	0.918	49.261	4.12	0.726	−0.382	−0.422
In this company, people protect their own interests above other considerations.	0.961	55.973	4.11	0.841	−0.998	1.046
**Feeling of guilt (Izard et al., 1974). (a = 0.957) (CR = 0.958, AVE = 0.884, MSV = 0.295)**						
Feeling guilty	0.958	b	3.85	1.024	−1.215	1.501
Feeling blameworthy	0.913	55.419	3.94	0.981	−1.440	2.304
Feeling repentant	0.949	64.608	3.91	.918	−1.083	1.710
**Customer-oriented citizenship behavior (Bettencourt and Brown, 1997) (a = 0.950) (CR = 0.979, AVE = 0.903, MSV = 0.462)**						
I voluntarily assisted customers even if it means going beyond job requirements	0.919	b	3.98	0.880	0.192	0.157
I helped customers with problems beyond what is expected required	0.965	60.541	3.95	0.919	0.340	0.157
I often went above and beyond the call of duty when serving customers	0.975	63.304	3.94	0.918	0.193	0.157
Today, I willingly went out of my way to make a customer satisfied	0.978	64.170	3.95	0.897	0.205	0.157
I frequently went out the way to help a customer	0.912	49.583	3.98	0.894	0.093	0.157
**Emotional exhaustion (Karatepe and Uludag, 2007; Maslach and Jackson, 1981) (a = 0.972) (CR = 0.970, AVE = 0.846, MSV = 0.335)**						
I feel emotionally drained from my work.	0.844	b	4.14	0.845	−0.891	0.529
I feel fatigued when I get up in the morning and have to face another day on the job.	0.840	33.990	4.14	0.860	−0.919	0.531
I feel burned out from my work.	0.930	41.127	4.02	1.019	−1.243	1.286
I feel frustrated by my job.	0.968	44.822	4.03	1.006	−1.237	1.332
I feel I am working too hard on my job.	0.967	44.733	4.00	1.048	−1.258	1.264
I feel like I am at the end of my rope	0.960	43.992	4.03	1.039	−1.309	1.470

Model fit: (χ^2^ (539, *N* = 970) = 1763.069, *p <* 0.001, normed χ^2^ = 3.271, RMSEA = 0.031, SRMR = 0.029, CFI = 0.954, TLI = 0.965, NFI = 0.961, PCFI = 0.745 and PNFI = 0.717). Note: CR = composite reliability, AVE = average variance extracted, MSV = maximum shared value. ^b^ Fixed to set the scales. M = Mean, S.D. = standard deviation.

**Table 3:** Discriminant validity employing the Fornell–Larcker criterion method.

	1	2	3	4	5	6	7
1—Emotional exhaustion	**0.920**						
2—UOB	0.468	**0.926**					
3—Job insecurity	0.334	0.407	**0.891**				
4—Work intensification	0.346	0.378	0.532	**0.949**			
5—Unethical company profit climate	0.579	0.524	0.426	0.502	**0.933**		
6—Feelings of guilt	0.523	0.530	0.343	0.469	0.543	**0.940**	
7—Customer-oriented citizenship behavior	0.423	0.390	0.499	0.680	0.515	0.511	**0.950**

Note: Bold diagonal numbers embody the Average Variance Extracted (AVEs) for the related factor.

**Table 4:** Result of research hypothesis.

Hypotheses		Results of Research Model		
		Beta (β)	C-R (t-Value)	Hypotheses Results
H1	Job insecurity → Unethical organization behavior	0.41 ***	12.187	Supported
H2	Work intensification → Unethical organization behavior	0.21 ***	7.226	Supported
H3	Unethical company-profit climate → Unethical organization behavior	0.44 ***	14.142	Supported
H4	Unethical organization behavior → Feeling of guilt	0.52 ***	17.000	Supported
H5	Unethical organization behavior → Customer-oriented citizenship behavior	0.39 ***	10.204	Supported
H6	Unethical organization behavior → Emotional exhaustion	0.46 ***	14.448	Supported

Model fit: (χ^2^ (554, N = 970) = 1865.872, *p* < 0.001, normed χ^2^ = 3.368, RMSEA = 0.038, SRMR = 0.047, CFI = 0.929, TLI = 0.931, NFI = 0.942, PCFI = 0.736 and PNFI = 0.731). Note: ***: Significant level less than 0.001.

## Data Availability

Data are available upon request from the researchers who meet the eligibility criteria. Kindly contact the first author privately through e-mail.
